# Systems for recognition and response to deteriorating emergency department patients: a scoping review

**DOI:** 10.1186/s13049-021-00882-6

**Published:** 2021-05-22

**Authors:** Julie Considine, Margaret Fry, Kate Curtis, Ramon Z. Shaban

**Affiliations:** 1 School of Nursing and Midwifery, Deakin University, Geelong, Victoria Australia; 2 Centre for Quality and Patient Safety Research, Deakin University, Geelong, Victoria Australia; 3 Institute for Health Transformation, Deakin University, Geelong, Victoria Australia; 4 Centre for Quality and Patient Safety Research, Eastern Health Partnership, Box Hill, Victoria Australia; 5 Faculty of Health, University of Technology Sydney, St Leonards, New South Wales Australia; 6 Northern Sydney Local Health District, St Leonards, New South Wales Australia; 7 Susan Wakil School of Nursing and Midwifery, The University of Sydney, Camperdown, New South Wales Australia; 8 Illawarra Shoalhaven Local Health District, Wollongong, New South Wales Australia; 9 Marie Bashir Institute for Infectious Diseases and Biosecurity, Faculty of Medicine and Health, The University of Sydney, Camperdown, New South Wales Australia; 10 Western Sydney Local Health District, Westmead, New South Wales Australia

**Keywords:** Emergency nursing, Emergency medicine, Emergency department, Rapid response team, Patient safety, Clinical deterioration, Deteriorating patients, Scoping review

## Abstract

**Background:**

Assessing and managing the risk of clinical deterioration is a cornerstone of emergency care, commencing at triage and continuing throughout the emergency department (ED) care. The aim of this scoping review was to assess the extent, range and nature of published research related to formal systems for recognising and responding to clinical deterioration in emergency department (ED) patients.

**Materials and methods:**

We conducted a scoping review according to PRISMA-ScR guidelines. MEDLINE complete, CINAHL and Embase were searched on 07 April 2021 from their dates of inception. Human studies evaluating formal systems for recognising and responding to clinical deterioration occurring after triage that were published in English were included. Formal systems for recognising and responding to clinical deterioration were defined as: i) predefined patient assessment criteria for clinical deterioration (single trigger or aggregate score), and, or ii) a predefined, expected response should a patient fulfil the criteria for clinical deterioration. Studies of short stay units and observation wards; deterioration during the triage process; system or score development or validation; and systems requiring pathology test results were excluded. The following characteristics of each study were extracted: author(s), year, design, country, aims, population, system tested, outcomes examined, and major findings.

**Results:**

After removal of duplicates, there were 2696 publications. Of these 33 studies representing 109,066 patients were included: all were observational studies. Twenty-two aggregate scoring systems were evaluated in 29 studies and three single trigger systems were evaluated in four studies. There were three major findings: i) few studies reported the use of systems for recognising and responding to clinical deterioration to improve care of patients whilst in the ED; ii) the systems for recognising clinical deterioration in ED patients were highly variable and iii) few studies reported on the ED response to patients identified as deteriorating.

**Conclusion:**

There is a need to re-focus the research related to use of systems for recognition and response to deteriorating patients from predicting various post-ED events to their real-time use to improve patient safety during ED care.

**Supplementary Information:**

The online version contains supplementary material available at 10.1186/s13049-021-00882-6.

## Introduction

Assessing and managing the risk of clinical deterioration is a cornerstone of emergency care, commencing at triage and continuing throughout the emergency department (ED) care [1]. EDs have improved patient outcomes through systematic approaches to assessment, risk management, and emergency care for specific patient groups, including trauma, stroke, sepsis and acute coronary syndrome [2–6]. For hospital patients, Rapid Response Systems (RRSs) provide guidance about recognising deteriorating patients and establish the standard for an expected response when deterioration occurs [7]. Systematic approaches for the recognition and response to clinical deterioration in ED, following the initial triage assessment have emerged over the last decade [8, 9].

Recognising and responding to clinical deterioration is an international patient safety priority and is an emergency care research priority [10–12]. The majority of serious in-hospital adverse events (unexpected death, cardiac arrest, unplanned intensive care admission) are preceded by vital sign abnormalities [13, 14]. Therefore, vital sign assessment, early recognition of vital sign abnormalities and an appropriate response is fundamental to effective emergency care and optimising patient outcomes. During ED care, 20 to 25% of patients have one or more abnormal vital signs [15, 16] and between 1.5 and 23% of ED patients experience clinical deterioration that fulfils ED-specific or hospital wide RRS activation criteria [15, 17–21]. Between 36 and 71% of ED adverse events are preventable [22] and up to one in seven ED patients have undetected clinical deterioration [15, 17, 18]. Failure to recognise and respond to clinical deterioration during emergency care increases high-mortality adverse events during emergency care and following the emergency care episode, irrespective of whether the patient is admitted to hospital or discharged [21, 23, 24].

The most common types of systems for recognising and responding to clinical deterioration are aggregate scoring or single trigger systems, and although their use has largely been reported in hospital inpatients [25, 26] their use in EDs is emerging in the literature [8, 9]. Aggregate scoring systems use the number and/or severity of physiological abnormalities to define the level of clinical deterioration and the corresponding escalation of care and expected response [26]. Single parameter systems are activated when any one of pre-defined criteria are fulfilled, and again there are different levels of criteria, linked to specific escalation of care processes and expected response [26]. In EDs, the use of both aggregate scoring systems [27] and single parameter systems have been reported [8, 20]. The aim of this scoping review was to assess the extent, range and nature of published research related to formal systems for recognising and responding to clinical deterioration in ED patients. The research questions addressed by this scoping review were: i) what are the formal systems for recognising and responding to clinical deterioration in emergency department (ED) patients; ii) how are these systems used? and iii) what is the impact of these systems on patient care and patient outcomes?

## Methods

### Study design

Given the heterogeneity of systems for recognising and responding to deteriorating patients, we elected to undertake a scoping review in preference to a systematic review. Scoping reviews have broader inclusion criteria compared to systematic reviews that have a narrow, clearly defined question [28]. In addition, the outcome of a scoping review is the volume of literature, types of studies conducted and the outcomes examined to date, rather than clinical outcomes that are typically examined in a systematic review so a scoping review was the most appropriate method to answer our question of interest. In scoping reviews, formal quality assessment is not usually performed and study findings are presented in a tabular format with accompanying narrative [29]. This scoping review was guided by the methodological framework developed by Arksey and O’Malley [30] (identify the research question; search for relevant studies; select studies; chart the data; collate, summarise, and report the results). The results were reported according to the Preferred Reporting Items for Systematic reviews and Meta-Analyses extension for scoping reviews (PRISMA-ScR) [31].

### Inclusion criteria

Studies were included if they were: human studies of ED patients (no age limits); published in English and original research evaluating formal systems for recognising and responding to clinical deterioration that occur after the triage assessment and that consisted of: i) predefined, documented patient assessment criteria for clinical deterioration (single trigger or aggregate score), and, or ii) a predefined, expected response for patients identified as deteriorating. Studies were excluded if they were: conducted in short stay units and ED observation wards (whether collocated in ED or not); related to recognising and responding to clinical deterioration during the triage process; system or score development or validation studies rather than studies of system use; or involved systems with criteria that required pathology test results. Editorials, letters, commentaries, opinion papers, case studies and case reports were also excluded.

### Search strategy

The following databases were searched on 7 April 2021: MEDLINE complete, Cumulative Index of Nursing and Allied Health literature (CINAHL) and Excerpta Medica database and (Embase). All databases were search from their inception with no date limiters (1966 MEDLINE; 1981 CINAHL and 1966 EMBASE). The full search strategy is available in Additional file 2.

### Study selection

The search results were downloaded into EndNote X8 and duplicates identified and removed. Two authors (JC and MF) independently screened titles and abstracts of studies against the inclusion criteria. Full text articles assessed as potentially eligible for inclusion in this review were independently screened against the inclusion criteria (JC and MF). Disagreements were resolved by discussion and consensus.

### Data extraction

Data were extracted by a single author (JC) and ratified by all co-authors. The characteristics of each study were extracted included the author(s), year of publication, study design, country, population, system tested, outcomes examined, and major findings. Human research ethics committee approval was confirmed for all included studies during the data extraction process.

## Results

**Fig. 1:**
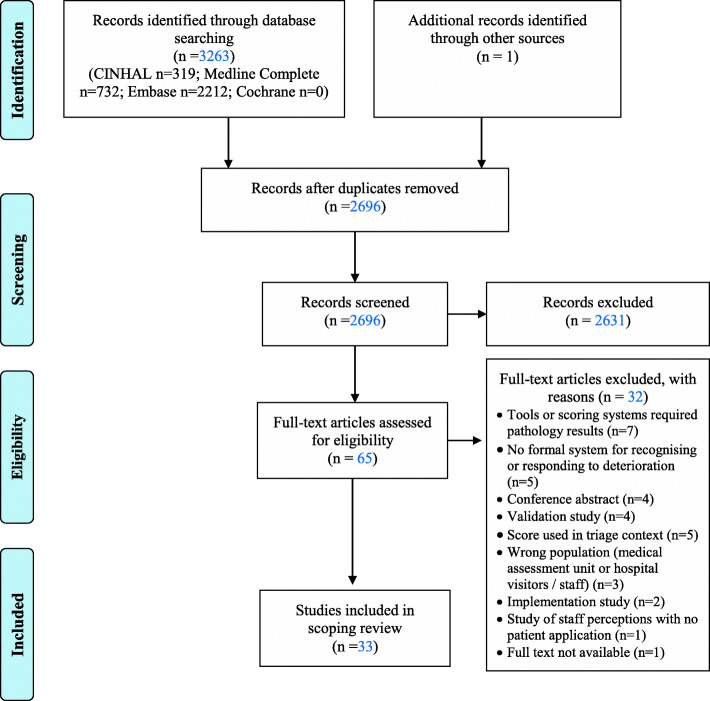
Preferred reporting items for systematic reviews and meta-analyses (PRISMA) diagram [31]

After removal of duplicates, our search returned 2696 publications and one additional publication was identified through hand searching. Of these, 2631 articles were excluded leaving 65 full text articles to be screened for eligibility (Fig. 1). Thirty-two articles were excluded because they involved systems requiring pathology test results (*n* = 7), were not a formal system for recognising or responding to deteriorating patients (*n* = 5), were using scores for triage (*n* = 5), were conference abstracts (*n* = 4), were tool validation studies (*n* = 4), focused on the wrong population (*n* = 3), described system implementation (*n* = 2) were a study of staff perceptions with no patient application (*n* = 1) or full text was not available (*n* = 1). In total, 33 studies representing 109,066 patients were identified for inclusion; all were observational studies. No additional studies of relevance were found by searching the grey literature or further hand-searching the reference lists of included articles.

### Study characteristics

A summary of the 33 included studies is presented in Supplementary Table 1. All studies were observational in nature, with 17 prospective studies [32–48] and 16 retrospective studies [19, 20, 49–62]. Thirty-two studies were single site studies [19, 20, 32–41, 43–62]. The countries of origin were Turkey (*n* = 8) [33, 34, 37–39, 42, 45, 48], United States (*n* = 3) [41, 43, 49], United Kingdom (*n* = 3) [44, 50, 51], Australia (*n* = 3) [19, 20, 57], China (*n* = 3) [35, 60, 62], Italy (*n* = 2) [58, 61], Indonesia (*n* = 2) [46, 47] and one study each from Finland [32], Demark [54], Norway [36], Netherlands [40]. Switzerland [55], Brazil [52], Singapore [53], Taiwan [59], and Korea [56].

Twenty-seven studies were of adults [19, 32–62], three of children [41, 43, 51] and three studies included both adults and children [20, 52, 54]. Specific patient groups studied were patients with COVID-19 [58, 61], community acquired infections [33], respiratory distress or dyspnoea [19, 36, 38], chest pain [19], abdominal pain [19], cancer [48], stroke [46], older ED patients ≥65 years [37, 56, 59], and trauma patients [52, 60, 62]. Seven studies purposefully targeted ED patients requiring hospital admission [32, 34, 45, 46, 56–58], two studies targeted patients requiring intensive care unit (ICU) admission [55, 59], and eight studies targeted patients triaged as having high levels of clinical urgency [35, 39, 40, 42, 50, 52–54].

### Recognition of clinical deterioration in ED patients

The systems for recognising clinical deterioration were highly variable (Supplementary Table 2). There were 22 aggregate scoring systems used in 29 studies [32–48, 50–54, 56–62] and three single trigger systems used in four studies [19, 20, 49, 55]. The most commonly used scoring systems were Modified Early Warning Score (MEWS) [33–35, 37–39, 42, 45, 46, 48, 52, 53, 57, 59–62] and National Early Warning Score (NEWS) [32, 36, 40, 47, 50, 56, 57, 61]. Only seven studies reported ongoing clinical assessment for deterioration during the patient’s ED care: four studies used single trigger systems [19, 20, 49, 55], two used MEWS [44, 46] and one study was of 13 different scoring systems [57]. In 14 studies, clinical assessment was reported on ED arrival only [32, 34–37, 42, 45, 50, 51, 53, 54, 56, 60, 61]. Clinical assessment was reported on ED arrival and one other time point in six studies (after first ED intervention [48], 15 min [58], 2 h [48], 6 hours [52] and ED discharge [43, 59]), ED arrival and two other time points in two studies (15 min and 30 minutes [38] and 1 h and ED discharge [40] and in one study clinical assessment was reported on ED discharge only [41].

### Response to clinical deterioration in ED patients

Only six studies explicitly reported the response to ED patients identified as deteriorating [19, 20, 44, 49, 52, 58]. Responses to single triggers included overhead page to prompt ED team to report to patient location [49], report to nurse and emergency physician in charge of the shift and patient medical review within 5 min [19, 20] and report to ED coordinator and doctor for review within 15 min and discuss management plan with specialist Registrar/Consultant within 30 min [44]. In one study, the physician was to be notified immediately when MEWS≥4 [52] and in another study, Novara COVID score was used to determine ED disposition decisions [58].

### Outcomes reported

Only two studies compared the absence and presence of a system for recognising and responding to deteriorating ED patients [19, 49]. In a pre-post test study, Imperato et al. [49] reported no significant difference in median hospital stay (3.8 vs 4.0 days, *p* = 0.21), special care unit stay (5.0 vs 5.6 days, *p* = 0.42), in-hospital mortality (6.0% vs 5.6%, *p* = 0.66) or frequency of upgrades in care level within 24 h (4.9% vs 4.0%, *p* = 0.52) following implementation of an ED trigger system. Considine et al. [19] showed a non-significant decrease in the frequency of unreported clinical deterioration following the staged implementation of ED clinical instability criteria, an escalation of care protocol, and track and trigger nursing observation charts.

#### Emergency department outcomes

Nine studies reported outcomes pertinent to the safety and quality of ED care (Table 1) [19, 20, 37, 38, 42, 44–46, 52]. Nine studies focused on the following patient outcomes: ED deaths [37, 42, 45, 52], resolution of clinical instability [20], ED treatment effectiveness [38], deterioration during ED care [46], early recognition of trauma severity to inform decisions regarding ICU or operating room transfer [52], and impact of clinical deterioration during ED care on ED length of stay [19]. Three studies focused on the ED processes of clinician use of systems for recognising and responding to deteriorating ED patients including system activation [20, 44], deterioration triggers, unreported clinical deterioration [19] and completeness of documentation [44].

**Table 1:** Emergency department outcomes

	Major findings
**Patient outcomes**	
**Deaths in ED (*n*** **= 4)** [37, 42, 45, 52]	Patients with a MEWS > 4, had higher odds of death in ED death than patients with MEWS< 4 (*p* < 0.001) [45]. Other studies reported conflicting results: patients who died in ED had
	• significantly higher MEWS (*p* < 0.001) and VIEWS (*p* < 0.001) scores [37]. or no significant differences in MEWS (*p* = 0.726) and REMS (*p* = 0.057) scores compared to patients discharged from ED [42].
	• significantly higher MEWS (*p* = 0.003) and VIEWS (*p* = 0.002) scores [37], or no significant differences in MEWS (0.306) and REMS (0.402) scores compared to patients admitted to the wards [42].
	• had no significant difference in MEWS (*p* = 0.130) and VIEWS (*p* = 0.058) scores [37] or MEWS (*p* = 0.810) and REMS (*p* = 0.626) scores compared to patients admitted to ICU [42].
	Of 115 patients studied by Rocha et al. two died in ED: both had a MEWS score of ≥7 at 6 h of ED care. No patients with MEWS 1, 2–3 or 4–6 at 6 h of ED care died (*p* < 0.001) [52].
**Clinical deterioration in ED** **(*n* = 1)** [46]	There was a significant correlation between MEWS and the risk of deterioration in the ED (AUC = 0.830, 95% CI 0.811–0.957, *p* < 0.001).
**Resolution of clinical instability/treatment effectiveness (*n*** **= 2)** [20, 38]	Median duration of clinical instability was 39 min (IQR, 5–129 min) and clinical instability was resolved in 64.2% of cases [20]. For patients with dyspnoea, there was a significant decrease in 30-min EWS (compared to 15-min EWS), which along with significant positive changes (towards return to normal) in all vital signs except temperature, indicating ED treatment effectiveness [38].
**Early recognition of trauma severity (*n*** **= 1)** [52]	Increasing MEWS during first 6 h of ED care had a significant relationship with transfer to the operating room, and ICU admission [52].
**ED length of stay (*n*** **= 1)** [19]	Patients who fulfilled ED clinical instability criteria during ED care had longer median ED length of stay than patients whose ED care was unaffected by clinical deterioration (7.2 h vs 4.4 h, *p* < 0.001) [19].
**Emergency department processes**	
**Clinician use of systems for recognising and responding to deteriorating ED patients (*n*** **= 3)** [19, 20, 44]	**System activation**
	A study of 204 patients with ED EWS activation showed that: 93.1% activations were by nurses and the median time between documenting physiological abnormalities and activation was 5 min (IQR: 0–20) [20]. Most patients who required an ED EWS activation, had one activation (91.7%), but 7.8% of patients had two activations and one patient (0.5%) had three activations during their ED care [20]. In a study of 472 ED episodes of care, 43.2% of ED patients had ≥1 escalation of care [44]: 56.8% of patients whose track & trigger score exceeded alert threshold had an escalation of care but 40.0% of patients whose track & trigger score did not exceed alert threshold still had escalation of care based presumably on clinician concern [44].
	**Deterioration triggers**
	Hypotension, tachycardia, bradycardia and tachypnoea were the most common reasons for ED EWS activation [20].
	The most common episodes of documented physiological abnormalities were tachypnoea (34%) followed by tachycardia (29%) and hypotension (17%) [19].
	**Unreported clinical deterioration during ED care**
	Unreported deterioration decreased with each stage of ED RRS implementation but was not statistically (*p* = 0.141) [19]:
	• Clinician discretion and no track and trigger charts (*n* = 150 patients): 86.7%
	• ED CIC and escalation of care protocol, no track and trigger charts (*n* = 150 patients): 68.8%
	• ED CIC and escalation of care protocol, new track and trigger charts (*n* = 150 patients): 55.3%
	• ED CIC and escalation of care protocol, 12 months after track and trigger charts (*n* = 150 patients): 54.0%
	**Completion of documentation**
	A study of 2965 sets of vital signs from 472 ED episodes of care showed 85.8% of patients had documentation of ≥1 complete set of six vital signs: 87.6% sets of vital signs contained HR, RR, BP and SpO_2_ and overall, 25.6% of vital signs were complete [44].
	**Track and trigger scoring**
	A total of 34.5% of observations (*n* = 2965 vital signs) contained a track & trigger score and 60.6% of patients had ≥1 track & trigger score documented in the ED [44]. However, 20.6% of track & trigger scores were incorrect, 79.1% of the incorrect track & trigger totals were underscored, and 93.4% of track & trigger score errors were from incorrect assignment of the score to an individual vital sign [44].

*ED* Emergency department, *MEWS* Modified Early Warning Score, *ViEWS* VitalPac Early Warning Score, *REMS* Rapid Emergency Medicine Score, *ICU* Intensive care unit, *AUC* Area under the receiver operating curve, *EWS* Early warning system, *CIC* Clinical instability criteria

#### Post emergency department outcomes

Twenty-seven studies reported in-patient outcomes occurring post the ED episode of care (Table 2). Two studies examined the effect of repeated assessment for deterioration during ED care *and* an expected response to patients identified as deteriorating, on outcomes occurring post ED discharge [19, 49]. These studies of patients who fulfilled clinical deterioration triggers and had internal escalation of care within the ED reported conflicting results. In one study, patients in whom this system was activated were more likely to require hospital admission or die in hospital than patients in whom this system was not activated [19]. The other study reported no difference in ICU admission, hospital length of stay or 30-day mortality following implementation of ED triggers and an escalation of care response [49].

**Table 2:** Post emergency care outcomes

Outcome	Major findings
**Studies** ***with*** **repeated clinical assessments during ED care** ***and*** **expected ED response to deteriorating patients**	
**Mortality (*n*** **= 2)**	Studies of ED trigger systems had conflicting results:
• In-hospital deaths (*n* = 1) [19]	• no difference in 30-day mortality after implementation of ED triggers [49], and
• 30 days (*n* = 1) [49]	• patients who fulfilled ED CIC during ED care were more likely to die in hospital than those who did not fulfil trigger criteria (6.3% vs 1.4%, *p* = 0.044) [19].
**ICU admission (*n*** **= 1)**	Implementation of ED triggers made no significant difference to days spent in special care units (ICU or intermediate care) [49].
• ICU cohort with no comparator [49]	
**Hospital admission (*n*** **= 10)**	Patients who fulfilled ED CIC during ED care were more likely to be admitted to hospital (72.7% vs 40.8%, *p* < 0.001) [19].
• Hospital admission vs discharge from ED [19]	
**Length of stay (*n*** **= 1)**	The introduction of ED triggers had no significant impact on hospital stay [49].
• Hospital length of stay [49]	
**Studies** ***without*** **repeated clinical assessments during ED care or an expected ED response to deteriorating patients**	
**Mortality (*n*** **= 22)**	The majority of studies of aggregate scoring systems showed that patients who died had higher scores than those who survived and this was the case for MEWS [34, 35, 37, 39, 42, 45, 48, 53], NEWS [32, 36, 40, 56, 61], NEWS2 [61], NEWS-C [61], qSOFA [33, 61], REMS [42, 61], GAP [39], BEWS [54], RTS [60] and Novara-COVID scores [58]. One study showed that MEWS progression (increasing scores) was associated with significantly higher 7- and 30-day mortality [59]. One study of a single trigger system applied at repeated intervals during ED care but with no specific response to deterioration reported showed that more patients who died in hospital fulfilled hospital MET criteria (applied in ED) compared to those who survived [55]. MEWS and RTS were good predictors of 24-h mortality, but MEWS had better predictive efficacy than RTS [60]. Most aggregate scoring systems were good or excellent predictors of 2-day in-hospital mortality but less predictive of in-hospital mortality at 7 and 28-days [57]. One study showed that REMS had the highest overall accuracy for 48-h and 7-day mortality compared to MEWs, NEWS, NEWS2, NEWS-C, and qSOFA [61].
• In-hospital deaths (*n* = 9) [32, 34, 35, 42, 45, 55, 56, 58]	
• 24 h (*n* = 1) [60]	
• 48 h (*n* = 3) [54, 57, 61]	
• 5 days (*n* = 3) [33, 36, 37], 7 days (*n* = 3) [57, 59, 61]	
• 14 days (*n* = 1) [33]	
• 28 days (*n* = 3) [33, 39, 57], 30 days (*n* = 7) [32, 36, 40, 48, 49, 53, 59]	
• 90 days (*n* = 1) [36]	
• Not reported (*n* = 1) [47]	
**ICU admission (*n*** **= 14)**	Patients admitted to ICU had higher aggregate scores than those not admitted to ICU and this was the case for MEWS [34, 35, 37, 42, 45], NEWS [32, 40], PEWS on ED arrival [43] and on ED discharge [41, 43], BEWS [54] and REMS [42] on ED arrival; NEWS at 1-h of ED care [40] and NEWS at ED discharge [40]. The majority (52.5%) of patients admitted to ICU had MEWS = 2–3 and no patient with MEWS ≥7 was admitted to ICU [52]. There was no significant difference in the proportion of patients admitted to ICU with MEWS≥4 versus MEWS < 4 [53]. Most aggregate scoring systems were poor predictors of need for ICU admission from wards within 2-days of hospital admission [57]. One study showed that NEWS had the highest overall accuracy for predicting ICU admission at both 48 h and 3 days, compared to MEWs, NEWS2, NEWS-C, qSOFa, and REMS [61].
• ICU admission compared to no ICU admission with not (*n* = 6) [34, 35, 40, 45, 52–54]	
• ICU admission compared to ward admission (*n* = 5) [32, 37, 41–43]	
• ICU admission compared to discharge from ED (*n* = 2) [37, 42]	
• ICU cohort with no comparator (*n* = 2) [52]	
• ICU admission from wards < 2 days of admission (*n* = 1) [57]	
• ICU admission within 48 h and 7 days of ED arrival [61]	
**Hospital admission (*n*** **= 10)**	Patients requiring hospital admission tended to have higher aggregate scores than those not admitted and this was the case for MEWS [35, 37, 48], NEWS [36, 40], VIEWS [37], PEWS [41, 51], on ED arrival; and EWS at 15 min and 30 min of ED care [38]. One study reported that patients admitted to hospital had significantly lower MEWS and REMS scores than patients discharged from ED or admitted to ICU [42]. One study reported no difference in the odds of hospital admission between patients with MEWS ≥4 versus MEWS < 4 [45].
• Hospital admission vs discharge from ED (*n* = 9) [35–38, 40–42, 45, 48, 51]	
• Hospital admission vs died in ED (*n* = 2) [37, 42]	
• Hospital admission vs ICU admission (*n* = 2) [37, 42]	
**Clinical stability (*n*** **= 3)**	Significantly lower levels of clinical stability occurred in patients with Novara-COVID scores of 3 (OR = 0.28, 95%CI 0.13–0.59) or 4–5 (OR = 0.03, 95%CI 0.006–0.12) [58]. One study showed that MEWS progression (increasing scores) was associated with significantly higher 24-h APACHE-II scores [59]. One study showed that severely injured patients had significantly higher MEWS and MEWS-A scores on ED arrival than less severely injured patients [62]. MEWS-A had greater predictive value than MEWS in identifying severely injured patients [62].
• No transfer to higher intensity of care (low to intermediate) and no in-hospital death (*n* = 1) [58]	
• Initial and 24-h ICU APACHE-II scores (*n* = 1) [59]	
• Injury severity (*n* = 1) [62]	
**Length of stay (*n*** **= 2)**	The hospital length of stay studies had conflicting results:
• Hospital length of stay (*n* = 2) [40, 55]	• NEWS ≥7 on ED arrival was associated with longer hospital length of stay [40], and
• ICU length of stay (*n* = 1) [55]	• no significant association between MET call criteria in the ED and hospital length of stay [55].
	There was no correlation between MET call criteria in the ED and ICU length of stay [55].

*ED* Emergency department, *CIC* Clinical instability criteria, *ICU* Intensive care unit, *EWS* Modified Early Warning Score, *NEWS* National Early Warning Score, *NEWS2* National Early Warning Score 2, *NEWS-C* Modified NEWS, *qSOFA* Quick Sepsis Related Organ Failure Assessment, *REMS* Rapid Emergency Medicine Score, *GAP* Glasgow Coma Scale-age-systolic blood pressure score, *BEWS* Bispebjerg Early Warning Score, *RTS* Revised Trauma Score, *ViEWS* VitalPac Early Warning Score, *PEWS* Pediatric Early Warning Score, *PEWS* Pediatric Early Warning Score, *APACHE II* Acute physiology and chronic health evaluation score, *MEWS-A* Modified early warning score with abdominal score, *MET* Medical emergency team

The remaining 25 studies reporting post ED outcomes lacked ongoing assessment for deterioration during ED care, an expected response to deteriorating patients, or both [32–43, 45, 51–59]. One study reported that patients had an average of nine MEWs score during ED care but did not report a process of care escalation for a high MEWs score [59]. One study reported using Novara-COVID scores at 15 min after ED arrival to inform ED discharge decisions (discharge or level of hospital care) but did not report repeated Novara-COVID score assessments during the remainder of ED care [58]. Mortality at a range of time points (in-hospital to 90 days) was the most commonly reported post-ED outcome (*n* = 22) (Table 2) [32–37, 39, 40, 42, 45, 47–49, 53–61]. ICU admission was reported in 14 studies (Table 2) [32, 34, 35, 37, 40–43, 45, 52–54, 57] and hospital admission was reported in ten studies (Table 2) [35–38, 40–42, 45, 51]. Three studies reported on clinical instability (Table 2): one was in the context of using MEWS to predict trauma severity [62], one was related to using Novara-COVID scores to inform ED disposition decisions [58] and one was related to the relationship between worsening MEWS in ED and risk of ICU mortality (acute physiology and chronic health evaluation scores) [59]. Two studies reported on hospital length of stay (Table 2): one reported that NEWS≥7 on ED arrival was associated with longer hospital stay [40], and one reported no association between fulfilling deterioration triggers in ED and length of hospital stay [55]. Finally, there was no correlation found between fulfilling MET triggers in ED and ICU length of stay [55] (Table 2).

## Discussion

This scoping review has identified and mapped the available evidence related to systems for recognition and, or response to clinical deterioration in ED patients. There were three major findings: i) few studies reported on how systems for recognising and responding to clinical deterioration were used to improve care of patients whilst in the ED; ii) the systems for recognising clinical deterioration in ED patients were highly variable; and iii) few studies reported on the ED response to patients identified as deteriorating.

The first major finding was that few studies reported on using systems for recognising and responding to clinical deterioration in ED patients to actually improve the quality and safety of emergency care. Only seven studies reported assessing for clinical deterioration on an ongoing basis or at repeated intervals during the patient’s ED care [19, 20, 44, 46, 49, 55, 57], so the clinical application of systems for recognition and response to clinical deterioration is poorly understood. Many studies in this review were framed as evaluating systems for recognising and, or responding to clinical deterioration, yet in 14 studies, clinical assessment occurred at a single point in time, most commonly ED arrival [32, 34–37, 42, 45, 50, 51, 53, 54, 56, 60, 61]. Clinical deterioration is defined as moving “from one clinical state to a worse clinical state which increases their individual risk of morbidity, including organ dysfunction, protracted hospital stay, disability, or death” [63] pp. 1031–1032. Therefore assessment of clinical status at one point in time cannot enable recognition of deterioration as such, and at best, can only enable recognition of a seriously ill patient. Consequently, future research should focus on real-time assessment for deterioration and appropriate escalation of care strategies during emergency care.

The ED is an assessment intensive environment where accurate measurement and interpretation of vital sign data is critical to the recognition of deteriorating ED patients, however there are few published studies of vital sign assessment in the ED context [15, 64]. Instead, the majority of studies in this scoping review used systems for recognising clinical deterioration in ED patients to predict events that occurred after ED discharge such as mortality, ICU admission, hospital admission, hospital or ICU length of stay [19, 32–43, 45, 48, 49, 51–61]. Thus, these studies were not measuring whether deterioration had or had not occurred but rather were measuring the risk of deterioration, usually beyond the ED episode of care. No studies reported implementing an intervention should the patient’s ED data be predictive of death, ICU admission or hospital admission.

The second major finding of this scoping review was the identification of 26 different systems for recognition of clinical deterioration in ED patients: across the 33 included studies, there were 22 different aggregate scoring systems and three single trigger systems. The clinical need for so many scoring systems is questionable, and whether they are superior to clinical judgement is unknown. A study of using in-patient physiological scoring systems in ED compared triage information from the Manchester Triage System MEWS, ASSIST score (Assessment Score for Sick Patient Identification & Step-Up in Treatment) and hospital MET criteria and concluded that physiological scoring systems would have “identified only a small number of additional patients as critically ill and added little to the triage system currently in use” [27].

Finally, only six studies reported a specific response to ED patients identified as deteriorating: two tested aggregate scoring systems [44, 52, 58] and three tested single trigger systems [19, 20, 49]. The most common responses were to notify emergency physicians, ED coordinators and ED nurse in-charge [19, 20, 44, 49, 52], however, only three studies reported an expected timeframe in which the response should occur [19, 20, 44]. ED responses to deteriorating patients are less developed than inpatient RRS that have a clear and expected response in terms of team composition and response timing [1, 7, 8]. There were 12 studies that were prospective in nature and used ED arrival data to determine an aggregate score but did not explicitly reported a response if the patient fulfilled specific criteria for clinical deterioration [32, 34–38, 40, 42, 43, 45, 46, 48].

The strengths of this review are the thorough and systematic search technique, clear inclusion and exclusion criteria, and comprehensive data extraction. The limitations of this review are that only publications in English were included. As this was a scoping review rather than a systematic review, risk of bias and quality assessments of the included studies were not performed [29]. There is a lack of high-certainty evidence as the published research to date has been mostly observational studies, many of which were single site cohort studies with limited sample sizes. The majority of the studies identified in this review were focused on predicting post-ED events highlighting a major gap in the research related to real-time recognition and response to deterioration in ED patients. Finally, there was significant heterogeneity across studies in terms of patient selection, patient characteristics, site selection, and outcomes examined, which at this point in time will likely preclude the capacity to undertake a systematic review with meta-analysis.

## Conclusion

The real-time use of systems to recognise and respond to clinical deterioration in ED patients and improve patient safety during their emergency care is poorly understood and should be a research priority. The usefulness of the plethora of different scoring systems for recognising deteriorating ED patients is questionable and research should focus on their clinical utility in recognising and responding to deteriorating ED patients, rather than their use in predicting post ED events. Responses to clinical deterioration in ED patients are poorly reported in the literature to date. Further, there remain major research gaps related to whether specific interventions in response to clinical deterioration in the ED improves patient outcomes or disrupts adverse events occurring after ED care. There is a need to re-focus the research related to use of systems for recognition and response to deteriorating ED patients from predicting various post-ED events to the real-time use of these systems to improve patient safety during their ED care.

## Supplementary Information

**Additional file 1: Supplementary Table 1.** Studies detailing systems for recognising and responding to clinical deterioration in emergency department patients. **Supplementary Table 2.** Systems for recognition of clinical deterioration in Emergency Department patients.

**Additional file 2.** Search strategy.
