# Supplementary material for: Systems for recognition and response to deteriorating emergency department patients: a scoping review
Source: Scand J Trauma Resusc Emerg Med. 2021 May 22;29:69. doi: 10.1186/s13049-021-00882-6 (PMC8140439; doi:10.1186/s13049-021-00882-6)
Supplement: Supplementary file 1 — Additional file 1: Supplementary Table 1. Studies detailing systems for recognising and responding to clinical deterioration in emergency department patients. Supplementary Table 2. Systems for recognition of clinical deterioration in Emergency Department patients. [file 13049_2021_882_MOESM1_ESM.pdf]

Supplementary Table 1: Studies detailing systems for recognising and responding to clinical deterioration in emergency department patients

Author, year, country of origin, study design	Aims	Population, sample size	Intervention (system(s) tested)	Timing of clinical assessment(s)	Response to patients identified as deteriorating	Outcomes (ED or inpatient)	Outcomes reported
**Single trigger systems**							
Imperato et al. 2017<br>USA<br>Retrospective, pre-post study (single site)	Determine if clinical triggers had an effect on hospital metrics	ED patients (≥18 years) meeting "trigger" criteria (n= 73965)	• ED triggers	Assessed at each vital sign assessment	Overhead page: patient's ED attending physician, resident, nurse, and technician report to patient location immediately	Inpatient	Length of hospital stay<br>Days in ICU or intermediate care unit<br>Mortality: in-hospital, 30-days<br>Upgrades in care level once admitted
Considine et al. 2015<br>Australia<br>Retrospective cross sectional study (single site)	Evaluate ED RRS on reporting of clinical deterioration<br>Examine differences between patients who did, and did not, deteriorate during ED care.	ED patients (≥18 years) shortness of breath, chest pain or abdominal pain (n=600)	• T0 2009: Clinician discretion<br>• T1 2010: ED clinical instability criteria (CIC) and escalation protocol vs<br>• T2 2011: ED CIC, escalation protocol plus track & trigger chart<br>• T3 2012: as per T2	At each patient assessment	Report to nurse and emergency physician in charge of the shift; patient review by emergency physician (or senior registrar) <5 minutes	Both	Unreported deterioration during ED care<br>ED length of stay<br>Hospital admission<br>Mortality: in-hospital
Considine et al. 2012<br>Australia<br>Retrospective cohort study (single site)	Evaluate uptake of ED early warning system for recognition of, and response to, clinical deterioration	Every 10th patient for whom the ED Early Warning System was activated (n=204: 188 adults and 16 children aged <16 years)	• Clinical instability criteria (ED CIC)	At each patient assessment	Report to nurse and emergency physician in charge of the shift; patient review by emergency physician (or senior registrar) <5 minutes	ED	Clinician uptake of ED early warning system<br>Resolution of clinical instability
Etter et al. 2008<br>Switzerland<br>Retrospective cohort study (single site)	To evaluate the early prognostic value of the MET calling criteria in patients admitted to ICU from ED	Consecutive adult patients admitted to ICU from ED (n=452)	• Hospital MET call criteria	ED arrival, at 0–15 minutes, 15 minutes to 1 hour, 1–2 hours, 2–4 hours, 4–8 hours, 8–24 hours	Not reported	Inpatient	Mortality: in-hospital<br>Mechanical ventilation<br>Hemodynamic instability vasopressors or inotropes during ICU stay)<br>Length of ICU stay, hospital stay

Supplementary Table 1: Studies detailing systems for recognising and responding to clinical deterioration in emergency department patients

Author, year, country of origin, study design	Aims	Population, sample size	Intervention (system(s) tested)	Timing of clinical assessment(s)	Response to patients identified as deteriorating	Outcomes (ED or inpatient)	Outcomes reported
**Aggregate scoring systems: adults**							
Gavelli et al. 2021<br>Italy<br>Retrospective cohort study (single site)	Evaluate the prognostic role of Novara-COVID score	ED patients (≥18 years) with confirmed COVID-19 (n=338) admitted via the ED	•Novara-COVID score	At 15 minutes after ED arrival	•0-1 point: consider discharge and home quarantine or low-intensity ward admission<br>•1-2 point(s): low-intensity ward admission<br>• 3 points: intermediate-intensity ward admission<br>•4-5 points: intensivist consult & ICU admission	Inpatient	Mortality: in-hospital<br>Clinical instability (transfer to a higher level of care)
Kao et al. 2021<br>Taiwan<br>Retrospective cohort study (single site)	Explore relationship between ED-MEWS progression & prognosis of elderly patients admitted ICU from ED	Consecutive older ED patients (≥ 65 years) with non-traumatic presentation admitted to ICU from ED (n=1423)	•MEWS	On ED arrival<br>On ED discharge<br>Critically ill patients = additional scores as clinically indicted (not defined)<br>Average of 9 MEWS during ED care	Not reported	Inpatient	Mortality: 7-day, 30-day<br>ICU APACHE-II score on ICU admission & 24-hours
Yu et al. 2021<br>China<br>Retrospective observational study (single site)	Assess the predictive value of MEWS & RTS for ED trauma patients who died within 24 hours	ED patients (≥ 16 years) with acute trauma (n=1739)	•MEWS<br>•RTS	On ED arrival	Not reported	Inpatient	Mortality: 24-hour
Amandaty et al. 2020<br>Indonesia<br>Prospective cohort study (single site)	Determine effectiveness of NEWS effectiveness a predictor of mortality in ED patients with heart failure	Adult ED patients with heart failure (n=64)	•NEWS	Not reported	Not reported	Not reported	Mortality: time point not reported

Supplementary Table 1: Studies detailing systems for recognising and responding to clinical deterioration in emergency department patients

Author, year, country of origin, study design	Aims	Population, sample size	Intervention (system(s) tested)	Timing of clinical assessment(s)	Response to patients identified as deteriorating	Outcomes (ED or inpatient)	Outcomes reported
Aygun et al. 2020<br>Turkey<br>Prospective cohort study (single site)	Evaluate predictive value of MEWS is in identifying critically ill ED patients with malignancy.<br>Determine whether initial ED treatment can change patient's MEWS which can predict mortality or hospital admission.	ED patients (≥18 years) with malignancy diagnosis (n=501)	•MEWS	On ED arrival<br>After initial ED treatment at 2nd hour of ED care	Not reported	Inpatient	Hospital admission<br>Mortality: 30-day
Covino et al. 2020<br>Italy<br>Retrospective observational study (single site)	Determine which ED EWS on ED arrival is most accurate at identifying adult COVID-19 patients at risk for ICU admission or death within seven days.	ED patients (≥18 years) with confirmed COVID-19 (n=334)	•MEWS<br>•NEWS<br>•NEWS2<br>•NEWS-C<br>•qSOFA<br>•REMS	On ED arrival only	Not reported	Inpatient	Mortality: 48-hours, 7-day<br>ICU admission: 48-hours, 7-day
Jiang et al. 2020<br>China<br>Retrospective cohort study (single site)	Evaluated & compare utility of MEWS & MEWS-A for identifying trauma severity (ISS≥16)	Consecutive adult ED patients with multiple trauma (n=1230)	•MEWS<br>•MEWS-A	Not reported	Not reported	Inpatient	Trauma severity (ISS<16 vs ISS≥16)
Kim et al. 2020<br>South Korea<br>Retrospective observational study (single site)	Determine if elevated NEWS at admission increases mortality in older patients admitted to the ED	Older ED patients (≥65 years) requiring hospital admission (n=3139)	•NEWS	On ED arrival only	Not reported	Inpatient	Mortality: in-hospital
Nafiah et al. 2019<br>Indonesia<br>Prospective observational study (single site)	Determine if MEWS can predict deterioration of patients with stroke in ED	ED patients (≥18 years) with stroke (n=109)	•MEWS	At each patient assessment	Not reported	ED	Patient deterioration (MEWS≥4)

Supplementary Table 1: Studies detailing systems for recognising and responding to clinical deterioration in emergency department patients

Author, year, country of origin, study design	Aims	Population, sample size	Intervention (system(s) tested)	Timing of clinical assessment(s)	Response to patients identified as deteriorating	Outcomes (ED or inpatient)	Outcomes reported
Spencer et al. 2019<br>Australia<br>Retrospective observational study (single site)	Determine which of 13 EWS best predicted important clinical outcomes	ED patients (≥18 years) requiring hospital admission (n=690)	•RAPS<br>•MEWS<br>•MEWS-GCS<br>•REMS<br>•Goodacre Score<br>•WPS<br>•Groarke Score<br>•ViEWS<br>•AbViEWS<br>•GAP<br>•VSS<br>•NEWS<br>•VSG score	At each patient assessment	Not reported	Inpatient	In-hospital mortality: 2-day, 7-day & 28-day<br>Clinical deterioration (cardiac arrest or ICU admission) within 2 days of admission
Beğenen et al. 2019<br>Turkey<br>Prospective observational study (single site)	Evaluate efficacy of the scoring systems used to determine the mortality of ED patients with infections	ED patients (≥18 years) with community acquired infections (n=400)	•MEWS<br>•qSOFA	Not reported	Not reported	Inpatient	Mortality: 5-days, 14-days and 28-days
Akgun et al. 2018<br>Turkey<br>Prospective observational study (single site)	Evaluate efficiency of MEWS & MEES in assessing disease severity and predicting mid term prognosis of patients admitted to hospital from ED	ED patients (≥18 years) requiring hospital admission (n=1051)	•MEWS<br>•MEES	On ED arrival only	Not reported	Inpatient	Disease severity<br>Mortality: in-hospital
Kivipuro et al. 2018<br>Finland<br>Prospective observational study (single site)	Investigate performance of NEWS to predict in-hospital and 30-day mortality in ED patients<br>Compare NEWS scores & outcomes of patients admitted to ICU directly from ED, admitted to ICU via hospital wards, not admitted to ICU	ED patients (≥18 years) requiring hospital admission (n=1354)	•NEWS	On ED arrival only	Not reported	Inpatient	Mortality: in-hospital, 30-days<br>ICU admission

Supplementary Table 1: Studies detailing systems for recognising and responding to clinical deterioration in emergency department patients

Author, year, country of origin, study design	Aims	Population, sample size	Intervention (system(s) tested)	Timing of clinical assessment(s)	Response to patients identified as deteriorating	Outcomes (ED or inpatient)	Outcomes reported
Xie et al. 2018<br>China<br>Prospective observational study (single site)	Evaluate MEWS performance in predicting in-hospital mortality of ED patients	ED patients (≥18 years) triaged as near death, critically ill or acute (n=383)	•MEWS	On ED arrival only	Not reported	Inpatient	Composite of in-hospital mortality and ICU admission<br>Hospital admission versus discharge patients
Bilben et al. 2016<br>Norway<br>Prospective observational study (single site)	Evaluate usefulness of NEWS in unselected adult ED patients with respiratory distress	ED patients (≥18 years) with respiratory distress (n=246).	•NEWS	On ED arrival only	Not reported	Inpatient	Survival: hospital discharge, 30-day, 90-day<br>Maximum level of care<br>Use of mechanical ventilation<br>Discharge destination
Dundar et al. 2016<br>Turkey<br>Prospective observational study (single site)	Evaluate MEWS and VIEWS in predicting hospitalization and in-hospital mortality in older ED patients	ED patients ≥65 years (n=671)	•MEWS<br>•ViEWS	On ED arrival only	Not reported	Inpatient	Hospital admission<br>Mortality: in ED, in-hospital
Karakaya et al. 2016<br>Turkey<br>Prospective observational study (single site)	Investigate whether EWS can identify patients at risk and predict treatment success in ED patients with dyspnoea.	ED patients (≥18 years) with dyspnoea (n=91)	•Early warning score	On ED arrival; at 15 minutes; at 30 minutes	Not reported	Both	Hospital admission or discharge<br>ED treatment effectiveness
Keep et al. 2016<br>United Kingdom<br>Retrospective observational study (single site)	Examine the relationship between initial ED NEWS and diagnosis of septic shock	ED patients (≥16 years), triaged as requiring: immediate treatment or treatment<10 minutes or treatment<60 minutes (n=500)	•NEWS	On ED arrival only	Not reported	Both	Diagnosis of septic shock
Köksal et al. 2016<br>Turkey<br>Prospective cohort study (single site)	Assess & compare GAP MEWS in predicting 4-week mortality in high urgency ED patients	ED patients (≥18 years) triaged requiring immediate treatment or treatment <10 minutes (n=502).	•MEWS<br>•GAP	Not reported	Not reported	Inpatient	Mortality: 4-weeks

Supplementary Table 1: Studies detailing systems for recognising and responding to clinical deterioration in emergency department patients

Author, year, country of origin, study design	Aims	Population, sample size	Intervention (system(s) tested)	Timing of clinical assessment(s)	Response to patients identified as deteriorating	Outcomes (ED or inpatient)	Outcomes reported
Rocha et al. 2016<br>Brazil<br>Retrospective cross-sectional study (single site)	Determine if MEWS is useful in early identification of trauma severity	ED patients (all ages) triaged as requiring treatment<10 minutes; who stayed in ED≥6 hours; and who had extremity trauma, traumatic brain injury, major trauma, or abdominal and thoracic trauma (n=115).	•MEWS	On ED arrival; at 6 hours	MEWS≥4 =physician should be informed immediately	ED	Early identification of clinical severity of trauma<br>Mortality: in ED
Alam et al. 2015<br>Netherlands<br>Prospective observational study (single site)	Explore performance of NEWS in predicting adverse outcomes & need for hospital admission in ED patients	ED patients (≥18 years) triaged as high risk or danger zone vital signs and not triaged to the resuscitation room (n=274).	•NEWS	On ED arrival; at 1 hour, at ED discharge	Not reported	Inpatient	ICU admission<br>Mortality: 30-days<br>Hospital admission<br>Hospital length of stay
Bulut et al. 2014<br>Turkey<br>Prospective observational cohort study (three sites)	Compare efficacy of MEWS & REMS in predicting in-hospital mortality and hospital admission in medical and surgical ED patients	ED patients ≥16 years and triaged as having life-threatening but treatable injuries requiring rapid medical attention or potentially life-threatening injuries, risk of organ loss, and cases with important rate of morbidity (n=2000)	•MEWS<br>•REMS	On ED arrival only	Not reported	Inpatient	Hospital admission vs discharge<br>ICU/HDU admission<br>Mortality: in ED, in-hospital

Supplementary Table 1: Studies detailing systems for recognising and responding to clinical deterioration in emergency department patients

Author, year, country of origin, study design	Aims	Population, sample size	Intervention (system(s) tested)	Timing of clinical assessment(s)	Response to patients identified as deteriorating	Outcomes (ED or inpatient)	Outcomes reported
Wilson et al. 2013 United Kingdom Prospective cohort study (single site)	Evaluate utilisation of paper-based track and trigger ED charts	ED patients (≥18 years) in the resuscitation room, 'majors', or observation ward (n=472 patients and n= 2965 vital sign observations).	•MEWS	At each vital sign assessment (minimum frequency=hourly). Triggers<br>• score of 3 in any category,<br>• total score of ≥4,<br>• decreased GCS by ≥2 points, OR<br>• non-physiological reason for escalation of care	If triggered, inform ED coordinator and doctor for review within 15 minutes; discuss management plan with specialist Registrar / Consultant within 30 minutes. If score decreasing, continue current management; if score static or increasing, consider critical care or specialist review	ED	Quality of care<br>• completion rates of paper documentation<br>• number of escalation events<br>• number of patients who had physiological escalations either on arrival or during their ED stay<br>• comparison of the T&T charts with the escalations (physiological or non-physiological) in care that occurred
Ho et al. 2013 Singapore Retrospective cohort study (single site)	Determine if MEWS is predictive of mortality & poor outcomes in critically ill ED patients	Critically ill ED patients (≥18 years) who required ECG monitoring; were triaged as requiring immediate treatment or non-ambulant, severe distress; and were recruited between 0800-1800 (n=1024).	•MEWS	On ED arrival only	Not reported	Inpatient	Mortality: in-hospital mortality up to 30-days<br>ICU or HDU admission from ED
Christensen et al. Denmark 2011 Retrospective cohort study (single site)	Evaluate BEWS to identify critically ill ED patients	Random sample of ED patients (age not specified) triaged as red (severely ill or injured) (n=162).	•BEWS	On ED arrival only	Not reported	Inpatient	Mortality: 48-hours<br>ICU admission within 48 hours<br>Critical illness (ICU admission or death within 48 hours of ED arrival)
Armagan et al. Turkey 2008 Prospective observational study (single site)	Evaluate if MEWS can predict ED death, hospital admission, ICU admission & in-hospital deaths	ED patients (age not specified) requiring hospital admission (n=309)	•MEWS	On ED arrival only<br>• MEWS >4 defined as high-risk	Not reported	Inpatient	Mortality: in ED, in-hospital<br>Hospital admission<br>ICU admission

Supplementary Table 1: Studies detailing systems for recognising and responding to clinical deterioration in emergency department patients

Author, year, country of origin, study design	Aims	Population, sample size	Intervention (system(s) tested)	Timing of clinical assessment(s)	Response to patients identified as deteriorating	Outcomes (ED or inpatient)	Outcomes reported
**Aggregate scoring systems: children**							
Lillitos et al. 2016<br>United Kingdom<br>Retrospective cohort study (single site)	Evaluate sensitivity & specificity Brighton PEWs & COAST PEWS in predicting hospital admission & significant illness	ED patients (aged 2 days – 17 years) (n=1921)	•PEWS<br>•COAST PEWS	On ED arrival only	Not reported	Both	Hospital admission<br>Detection of significant illnesses
Breslin et al. 2014<br>USA<br>Prospective observational study (single site)	Determine association between ED discharge PEWS and level of hospital care	ED patients aged 0 to 21 years (n=383)	•PEWS	On ED discharge only	Not reported	Inpatient	Level of care at ED discharge: discharge from ED, acute care unit admission or ICU admission
Gold et al. 2014<br>USA<br>Prospective observational study (single site)	Test characteristics of ED PEWS score for ICU admission or ward clinical deterioration patients admitted from ED	ESI category 2 or 3 ED patients aged 0-21 years (n= 12,306) admitted to wards or ICU from ED	•PEWS	On ED arrival; at time of admission	Not reported	Inpatient	ICU admission (from ED or from the ward within 24 hours of admission)<br>Ward admission with no ICU transfer

AbViEWS = abbreviated VitalPac Early Warning Score; BEWS = Bispebjerg Early Warning Score; CIC = clinical instability criteria; ED = emergency department; ESI = Emergency Severity Index; GAP = Glasgow Coma Scale-age-systolic blood pressure score; GCS = Glasgow Coma Score; HDU = high dependency unit; ICU = intensive care unit; ISS = injury severity score; MEES = Mainz Emergency Evaluation Score; MET = medical emergency team; MEWS = modified early warning score; MEWS-A = modified early warning score with abdominal score; MEWS-GCS = Modified Early Warning Score with Glasgow Coma Score; MREMS = Rapid Emergency Medicine Score; NEWS = National Early Warning Score; NEWS2 = National Early Warning Score 2; NEWS-C = Modified NEWS; PEWS = Pediatric Early Warning Score; qSOFA= Quick Sepsis Related Organ Failure Assessment; RAPS = Rapid Acute Physiology Score; REMS = Rapid Emergency Medicine Score; RRS = rapid response system; RTS = Revised Trauma Score; USA = United States of America; ViEWS = VitalPac Early Warning Score; VSG = Vital Sign Group; VSS = Vital Signs Score; WPS = Worthing Physiological Score;

Supplementary Table 2: Systems for recognition of clinical deterioration in Emergency Department patients

	Age	Airway	Respiratory rate	Oxygen saturation	Heart rate	Heart Rhythm	Blood pressure	Temperature	Conscious state	Pain	Supplemental oxygen	Urine output	Concern	Capillary refill	Skin color	Respiratory effort	Co-morbidity
**Aggregate scoring systems**																	
Modified Early Warning Score (MEWS / MEWS-A^) [33 34 35 37-39 42 44 45 46 48 52 53 57 59-62]	No	No	Yes	Yes	Yes	No	Yes	Yes	AVPU	No	No	No	No	No	No	No	No
MEWS with Glasgow Coma Score (GCS) [57]	No	No	Yes	Yes	Yes	No	Yes	Yes	GCS	No	No	No	No	No	No	No	No
National Early Warning Score (NEWS / NEWS2 / NEWS-C^^) 32 [36 40 47 50 56 57 61]	No	No	Yes	Yes	Yes	Yes	Yes	Yes	AVPU	No	Yes	No	No	No	No	No	No
Bispebjerg Early Warning Score (BEWS) [54]	No	No	Yes	No	Yes	No	Yes	Yes	AVPU	No	No	No	No	No	No	No	No
Mainz Emergency Evaluation Score (MEES) [33]	No	No	Yes	Yes	Yes	Yes	Yes	No	GCS	Yes	No	No	No	No	No	No	No
Pediatric Early Warning Score (PEWS) [41 43]	No	No	Yes	No	No	No	No	No	Behaviour	No	Yes	No	No	Yes	Yes	Accessory muscle use	No
Brighton Pediatric Early Warning Score (PEWS) [51]	No	No	Yes	No	Yes	No	No	No	Yes	No	Yes	No	Clinician or family	No	No	Moderate-severe respiratory distress	No
COAST Pediatric Early Warning Score (PEWS) [51]	No	No	Yes	Yes	Yes	No	No	No	Yes	No	No	No	Clinician or family	No	No	Moderate-severe respiratory distress	No
Rapid Emergency Medicine Score (REMS) [42 57 61]	Yes	No	Yes	Yes	Yes	No	Yes (MAP)	No	GCS	No	No	No	No	No	No	No	No
Revised Trauma Score (RTS)[60]	No	No	Yes	No	No	No	Yes	No	GCS	No	No	No	No	No	No	No	No
VitalPac Early Warning Score (ViEWS) [37 57]	No	No	Yes	Yes	Yes	No	Yes	Yes	AVPU	No	Yes	No	No	No	No	No	No

Supplementary Table 2: Systems for recognition of clinical deterioration in Emergency Department patients

	Age	Airway	Respiratory rate	Oxygen saturation	Heart rate	Heart Rhythm	Blood pressure	Temperature	Conscious state	Pain	Supplemental oxygen	Urine output	Concern	Capillary refill	Skin color	Respiratory effort	Co-morbidity
Abbreviated VitalPac Early Warning Score (AbViEWS) [57]	No	No	Yes	Yes	Yes	No	Yes	Yes	No	No	Yes	No	No	No	No	No	No
Track & trigger[# 44]	Yes	No	Yes	Yes	Yes	No	Yes	Yes	AVPU	Yes	No	No	Patient or family	No	Yes	No	No
Quick Sepsis Related Organ Failure Assessment (qSOFA) [32 61]	No	No	Yes (>22/min)	No	No	No	Yes (SBP ≤100mm Hg)	No	GCS (<15)	No	No	No	No	No	No	No	No
Glasgow Coma Scale-age-systolic blood pressure (GAP) score [39 57]	Yes	No	No	No	No	No	Yes	No	GCS	No	No	No	No	No	No	No	No
Rapid Acute Physiology Score (RAPS) [57]	Yes	No	Yes	Yes	Yes	No	Yes	No	GCS	No	No	No	No	No	No	No	No
Goodacre Score [57]	Yes	No	No	No	Yes	No	No	No	GCS	No	No	No	No	No	No	No	No
Worthing Physiological Score (WPS) [57]	No	No	Yes	Yes	Yes	No	Yes	Yes	AVPU	No	No	No	No	No	No	No	No
Groarke Score [57]	No	No	Yes	No	Yes	No	Yes	Yes	AVPU	No	Yes	No	No	No	No	No	No
Vital Signs Score (VSS) [57]	No	Yes	Yes	Yes	Yes	No	Yes	No	GCS Seizures	No	No	No	No	No	No	No	No
Vital Sign Groups Score [57]	No	No	Yes	Yes	Yes	No	Yes	Yes	GCS	No	No	No	No	No	No	No	No
Novara-COVID Score[58]	No	No	Yes	Yes	No	No	No	No	No	No	No	No	No	No	No	No	Yes
**Single trigger systems**																	
ED Clinical Instability Criteria (adults) [19 20]	No	Yes	Yes (<10 or >30/min)	Yes (<90% on oxygen 10L/min)	Yes (<50 or >120 /min)	No	Yes (SPB <90 or >200 mmHg)	No	GCS Seizures	No	No	Yes (<20ml/ hour or <100 ml/ 6 hours)	Clinician	No	No	No	No
ED Clinical Instability Criteria (paediatric) [19 20]	No	Yes	Yes (tachypnoe a for age)	Yes (<90% on oxygen 10L min)	Yes (brady-cardia / tachy-cardia for age)	No	Yes	No	GCS Seizures	No	No	No	Clinician	No	No	No	No

Supplementary Table 2: Systems for recognition of clinical deterioration in Emergency Department patients

	Age	Airway	Respiratory rate	Oxygen saturation	Heart rate	Heart Rhythm	Blood pressure	Temperature	Conscious state	Pain	Supplemental oxygen	Urine output	Concern	Capillary refill	Skin color	Respiratory effort	Co-morbidity
Medical Emergency Team criteria [55]	No	Yes	Yes (6 or >36 /min)	Yes (<90% on oxygen)	Yes (<40 or >140 /min)	No	Yes (SBP<90 mmHg)	No	Yes (GCS<13 or drop by ≥2 points) Seizures	No	No	No	Clinician	No	No	No	No
ED triggers [49]	No	No	Yes (<8 or >30/min)	Yes (<90% on room air)	Yes (<40 or >130 /min)	No	Yes (SBP<90 mmHg)	No	No	No	No	No	No	No	No	No	No

AVPU=alert, responds to voice, responds to pain, unresponsive; /min=pre minute; SBP=systolic blood pressure; GCS=Glasgow Coma Score; L=litre

^ MEWS-A = MEWS with addition of scores for abdominal or thoracic tenderness, abdominal rigidity, flail chest

^^ NEWS-C = NEWS with the addition of a score for a confirmed history of chronic obstructive pulmonary disease

Track & trigger non-physiological reason for escalation of care: airway compromise, blood loss, systemic or extremity skin colour change, loss of movement or weakness of arm, leg or face, vomiting, unresolved or new pain, self-harm in ED, concern re ED LOS, patient / relative request
