# Supplementary material for: Systems for recognition and response to deteriorating emergency department patients: a scoping review
Source: Scand J Trauma Resusc Emerg Med. 2021 May 22;29:69. doi: 10.1186/s13049-021-00882-6 (PMC8140439; doi:10.1186/s13049-021-00882-6)
Supplement: Supplementary file 2 — Additional file 2. Search strategy. [file 13049_2021_882_MOESM2_ESM.docx]

**Additional file 2: SEARCH STRATEGY**

**Date: inception (1966) to 07 April 2021**

**Database: MEDLINE Complete via Ebsco**

**Time: unlimited**

**Limiters: published in English, human studies**

| **#** | **Query** |  |
| --- | --- | --- |
| **S26** | **S9 AND S25** | **732** |
| **S25** | **S10 OR S11 OR S12 OR S13 ORS14 OR S15 OR S16 OR S17 ORS18 OR S19 OR S20 OR S21 ORS22 OR S23 OR S24** | **7,992** |
| S24 | (MH "Hospital Rapid Response Team+") | 864 |
| S23 | (MH “Clinical Deterioration+”) | 367 |
| S22 | TI “between the flags” OR AB “between the flags” | 15 |
| S21 | TI “deteriorating patient*” OR AB “deteriorating patient*” | 474 |
| S20 | TI “clinical* deteriorat*” OR AB “clinical* deteriorat*” | 3,756 |
| S19 | TI “track and trigger” OR AB “track and trigger” | 93 |
| S18 | TI “modified early warning score” OR AB “modified early warning score” | 195 |
| S17 | TI “national early warning score” OR AB “national early warning score” | 241 |
| **S16** | TI “early warning s*” OR AB “early warning s*” | 2,560 |
| S15 | TI "patient at risk team*" OR "patient at risk team*" | 6 |
| S14 | TI "patient at-risk team*" OR AB "patient at-risk team*" | 6 |
| S13 | TI “rapid response system*” OR AB “rapid response system*” | 303 |
| S12 | TI “rapid response team*” OR AB “rapid response team*” | 675 |
| S11 | TI “critical care outreach” OR AB “critical care outreach” | 108 |
| S10 | TI “medical emergency team*” OR AB “medical emergency team*” | 488 |
| **S9** | **S1 OR S2 OR S3 OR S4 OR S5OR S6 OR S7 OR S8** | **129,582** |
| S8 | (MH "Emergency Medicine+”) | 11,926 |
| S7 | (MH "Emergency Nursing+") | 6,698 |
| S6 | (MH “Emergency Medical Services+”) | 73,750 |
| S5 | TI “emergency nursing” OR AB “emergency nursing” | 470 |
| S4 | TI “emergency medicine” OR AB “emergency medicine” | 10,137 |
| S3 | TI “accident and emergency” OR AB “accident and emergency” | 3,809 |
| S2 | TI "emergency room" OR AB "emergency room" | 12,966 |
| S1 | TI "emergency department" OR AB "emergency department” | 67,305 |

**Date: inception (1981) to 07 April 2021**

**Database: CINAHL Complete via Ebsco**

**Time: unlimited**

**Limiters: published in English, human studies**

| **#** | **Query** |  |
| --- | --- | --- |
| **S26** | **S9 AND S25** | **319** |
| **S25** | **S10 OR S11 OR S12 OR S13 ORS14 OR S15 OR S16 OR S17 ORS18 OR S19 OR S20 OR S21 ORS22 OR S23 OR S24** | **2,522** |
| S24 | (MH "Hospital Rapid Response Team") | 320 |
| S23 | (MH “Clinical Deterioration”) | 337 |
| S22 | TI “between the flags” OR AB “between the flags” | 6 |
| S21 | TI “deteriorating patient*” OR AB “deteriorating patient*” | 236 |
| S20 | TI “clinical* deteriorat*” OR AB “clinical* deteriorat*” | 768 |
| S19 | TI “track and trigger” OR AB “track and trigger” | 40 |
| S18 | TI “modified early warning score” OR AB “modified early warning score” | 113 |
| S17 | TI “national early warning score” OR AB “national early warning score” | 98 |
| S16 | TI “early warning s*” OR AB “early warning s*” | 801 |
| S15 | TI "patient at risk team*" OR "patient at risk team*" | 3 |
| S14 | TI "patient at-risk team*" OR AB "patient at-risk team*" | 3 |
| S13 | TI “rapid response system*” OR AB “rapid response system*” | 146 |
| S12 | TI “rapid response team*” OR AB “rapid response team*” | 325 |
| S11 | TI “critical care outreach” OR AB “critical care outreach” | 64 |
| S10 | TI “medical emergency team*” OR AB “medical emergency team*” | 226 |
| **S9** | **S1 OR S2 OR S3 OR S4 OR S5OR S6 OR S7 OR S8** | **98,820** |
| S8 | (MH "Emergency Medicine+”) | 12,825 |
| S7 | (MH "Emergency Nursing+") | 15,528 |
| S6 | (MH “Emergency Service+”) | 63,703 |
| S5 | TI “emergency nursing” OR AB “emergency nursing” | 329 |
| S4 | TI “emergency medicine” OR AB “emergency medicine” | 3,581 |
| S3 | TI “accident and emergency” OR AB “accident and emergency” | 1,163 |
| S2 | TI "emergency room" OR AB "emergency room" | 3,058 |
| S1 | TI "emergency department" OR AB "emergency department” | 27,195 |

**Date: inception (1966) to 07 April 2021**

**Database: EMBASE via EMBASE.com**

**Time: unlimited**

**Limiters: published in English, human studies**

| **S27** | **#9 AND #26** | **2,212** |
| --- | --- | --- |
| **S26** | **#10 OR #11 OR #12 OR #13 OR #14 OR #15 OR #16 OR #17 OR #18 OR #19 OR #20 OR #21 OR #22 OR #23 OR #24 OR #25** | **48,228** |
| S25 | 'rapid response team'/exp AND [humans]/lim AND [english]/lim | 2,616 |
| S24 | 'deterioration'/exp AND [humans]/lim AND [english]/lim | 40,297 |
| S23 | 'between the flags':ab,ti AND [humans]/lim AND [english]/lim AND [embase]/lim | 17 |
| S22 | 'deteriorating patient*':ab,ti AND [humans]/lim AND [english]/lim AND [embase]/lim | 605 |
| S21 | 'clinical deteriorat*':ab,ti AND [humans]/lim AND [english]/lim AND [embase]/lim | 6,440 |
| S20 | 'track and trigger':ab,ti AND [humans]/lim AND [english]/lim AND [embase]/lim | 107 |
| S19 | 'modified early warning score':ab,ti AND [humans]/lim AND [english]/lim AND [embase]/lim | 345 |
| S18 | 'national early warning score':ab,ti AND [humans]/lim AND [english]/lim AND [embase]/lim | 433 |
| S17 | 'early warning system*':ab,ti AND [humans]/lim AND [english]/lim AND [embase]/lim | 1,185 |
| S16 | 'early warning score*':ab,ti AND [humans]/lim AND [english]/lim AND [embase]/lim | 1,438 |
| S15 | 'patient at risk team*':ab,ti AND [humans]/lim AND [english]/lim AND [embase]/lim | 3 |
| S14 | 'patient at-risk team*':ab,ti AND [humans]/lim AND [english]/lim AND [embase]/lim | 3 |
| S13 | 'rapid response system*':ab,ti AND [humans]/lim AND [english]/lim AND [embase]/lim | 357 |
| #12 | 'rapid response team*':ab,ti AND [humans]/lim AND [english]/lim AND [embase]/lim | 966 |
| #11 | 'critical care outreach':ab,ti AND [humans]/lim AND [english]/lim AND [embase]/lim | 176 |
| #10 | 'medical emergency team*':ab,ti AND [humans]/lim AND [english]/lim AND [embase]/lim | 547 |
| **#9** | **#1 OR #2 OR #3 OR #4 OR #5 OR #6 OR #7 OR #8** | **210,447** |
| #8 | 'emergency medicine'/exp AND [humans]/lim AND [english]/lim | 32,088 |
| #7 | 'emergency nursing'/exp AND [humans]/lim AND [english]/lim | 6,218 |
| #6 | 'emergency ward'/exp AND [humans]/lim AND [english]/lim | 147,350 |
| #5 | 'emergency nursing':ab,ti AND [humans]/lim AND [english]/lim AND [embase]/lim | 93 |
| #4 | 'emergency medicine':ab,ti AND [humans]/lim AND [english]/lim AND [embase]/lim | 15,837 |
| #3 | 'accident and emergency':ab,ti AND [humans]/lim AND [english]/lim AND [embase]/lim | 3,667 |
| #2 | 'emergency room':ab,ti AND [humans]/lim AND [english]/lim AND [embase]/lim | 25,168 |
| #1 | 'emergency department':ab,ti AND [humans]/lim AND [english]/lim AND [embase]/lim | 111,181 |
